# LBD1 of Vitellogenin Receptor Specifically Binds to the Female-Specific Storage Protein SP1 via LBR1 and LBR3

**DOI:** 10.1371/journal.pone.0162317

**Published:** 2016-09-16

**Authors:** Lina Liu, Yejing Wang, Yu Li, Ying Lin, Yong Hou, Yan Zhang, Shuguang Wei, Peng Zhao, Ping Zhao, Huawei He

**Affiliations:** 1 State Key Laboratory of Silkworm Genome Biology, Southwest University, Beibei, Chongqing, 400715, China; 2 Chongqing Engineering and Technology Research Center for Novel Silk Materials, College of Biotechnology, Southwest University, Beibei, Chongqing, 400715, China; Tsinghua University School of Life Sciences, CHINA

## Abstract

Storage proteins are the major protein synthesized in the fat body, released into hemolymph and re-sequestered into the fat body before pupation in most insect species. Storage proteins are important amino acid and nutrition resources during the non-feeding pupal period and play essential roles for the metamorphosis and oogenesis of insects. The sequestration of storage protein is a selective, specific receptor-mediated process. However, to date, the potential receptor mediating the sequestration of storage protein has not been determined in *Bombyx mori*. In this study, we expressed and purified the first ligand binding domain of *Bombyx mori* vitellogenin receptor (BmVgR), LBD1, and found LBD1 could bind with an unknown protein from the hemolymph of the ultimate silkworm larval instar *via* pull-down assay. This unknown protein was subsequently identified to be the female-specific storage protein SP1 by mass spectrometry. Furthermore, far western blotting assay, immunoprecipitation and isothermal titration calorimetry analysis demonstrated LBD1 specifically bound with the female-specific SP1, rather than another unisex storage protein SP2. The specific binding of LBD1 with SP1 was dependent on the presence of Ca^2+^ as it was essential for the proper conformation of LBD1. Deletion mutagenesis and ITC analysis revealed the first and third ligand binding repeats LBR1 and LBR3 were indispensable for the binding of LBD1 with SP1, and LBR2 and LBR4 also had a certain contribution to the specific binding. Our results implied BmVgR may mediate the sequestration of SP1 from hemolymph into the fat body during the larval-pupal transformation of *Bombyx mori*.

## Introduction

A feature of holometabolous insects is undergoing complete morphological changes during their entire life cycle. Metamorphosis occurs during the transformation from a larva to a non-feeding pupa involves complete reconstructing of the insect and its tissues. At the end of the last larval stage, numerous proteins such as storage proteins (SPs) [1] are synthesized as the building blocks required for the formation of pupa, as pupa no longer can take up food and nutritions from external source. Storage proteins are synthesized abundantly during the active feeding larval period in the larval fat body, and subsequently secreted into the hemolymph. These proteins are sequestered from the hemolymph into the fat body prior to pupation and stored as dense crystalline granules until they are proteolytically hydrolyzed to supply the necessary amino acids for pupal development [2–4]. The sequestration and utilization of storage proteins not only allows holometabolous insects to survive during the non-feeding period of pupa, but also enables their diverse strategies of reproduction.

Most known storage proteins belong to hexamerins family [5]. Hexamerins have been identified in almost all insect species, which are composed of six identical or similar subunits with an average molecular weight of 80 kDa [6, 7]. Hexamerin subunits are generally classified into three major classes based on their biochemical properties: 1) arylphorins or aromatic-rich proteins (74–80 kDa) [8, 9]; 2) female-specific methionine-rich proteins (82 kDa) [10]; and 3) small molecules specific binding hexamerins [4, 11]. Two types of storage proteins, SP1 and SP2 have been identified in the larval hemolymph of *Bombyx mori*. SP1 is a methionine-rich protein, and SP2 is an aromatic-rich protein. SP1 is massively synthesized in the fat body of female larva during the last larval instar, then secreted into hemolymph and sequestered into the fat body shortly before pupation [10, 12–14]. The uptake of storage protein by the insect larval fat body is a stage-specific event occurring only shortly before pupation, and this is a selective, receptor-mediated endocytotic process [4, 15, 16], similar to the transportion of the yolk protein vitellogenin (Vg) mediated by its specific receptor termed as VgR.

Vitellogenin is a precursor of egg yolk protein specifically expressed in the fat body of the females in nearly all oviparous species.The endocytosis of Vg mediated by VgR is essential for the metamorphosis and oogenesis of oviparous species [17]. VgR belongs to the low density lipoprotein receptor (LDLR) superfamily [18, 19]. LDLR members bind to a variety of ligands and are widely involved in the amino acid and lipid metabolism in both vertebrates and invertebrates [20]. These receptors share a number of common structural features, including (i) ligand-binding domain (LBD), (ii) epidermal growth factor precursor (EGFP) homology domain, (iii) O-linked sugar domain, (iv) a single transmembrane domain and (v) a cytoplasmatic domain. Classical LBD consists of numbers of class A cysteine-rich ligand binding repeats (LBRs), and EGFP is composed of class B cysteine-rich repeats and YWXD repeats [21, 22]. *Nilaparvata lugens* VgR contains two LBDs with five LBRs in LBD1 and eight LBRs in LBD2 [23], however, *Spodoptera litura* [24] and *Bombyx mori* [25] only have four LBRs in LBD1 and seven LBRs in LBD2. Some large receptors such as LDLR-related protein and megalin even have more than 30 LBRs in several clusters [26, 27]. Each LBR consists of about 40 amino acid residues, which include 6 cysteine residues (CI-VI). These cysteine residues pair in such a way of CI-CIII, CII-CV and CIV-CVI to form three disulfide bonds, which thus facilitate the proper folding and stabilize the conformation of LBD [28, 29]. LDLR members could bind with a variety of un-related ligands [30, 31]. The specificity and multiplicity of LDLR binding with its ligand are mainly determined by the numbers and the arrangement of LBRs in LBD [32, 33]. LBRs are not functionally identical, although their sequences are highly conserved [34, 35]. For instance, LBR3 of chicken VgR is important for the binding of LBD with the receptor-associated protein (RAP) [36], however, mutational analysis suggests LBR5 is critical for the binding of apoE with LDLR, and LBR2-6 is required for the binding of apoB. Any two adjacent LBRs are required at least for the binding of LBD with RAP [37].

Vg and SP1 are the major proteins synthesized in the fat body and released into the hemolymph of female insects [38]. The endocytosis of Vg mediated by VgR has been well documented [19], it is still uncertain whether the receptor is present to mediate the sequestration of storage proteins in *Bombyx mori*, although numbers of storage protein receptors have been identified and well characterized in the Lepidopteran species *Helicoverpa zea* and *Corcyra cephalonica* [39, 40], *Calliphora vicina* [41], and Diptera insects [42, 43]. Because of the physiological significance of storage proteins on the development of *Bombyx mori*, the aim of this study was to provide conclusive evidence for the receptor-mediated endocytosis of storage proteins in *Bombyx mori* through the detailed biochemical analysis of the interactions between LBD1 of VgR and storage proteins.

## Materials and Methods

### Biological materials

*Bombyx mori* strain P50 (Dazao) was reared as previously described [44]. Hemolymph was collected from female silkworm larvae on day 7 of the fifth instar.

### Bioinformatical analysis

*BmVgR* gene (ID: NM_001197251) structure has been determined [25]. Signal peptide was predicted by SignalP 4.1 (http://www.cbs.dtu.dk/services/SignalP/). Multiple sequence alignment was performed using Clustal X [45] and rendered by ESPript 3.0 [46].

### Expression vector construction of LBD1 and its mutants

LBD1 and its mutants were constructed as follows: LBD1 (Q_1_-H_199_), LBD1-ΔR1 (C_58_-H_199_), LBD1-ΔR2 (Q_1_-F_57_-K_103_-H_199_), LBD1-ΔR3 (Q_1_-K_103_-C_154_-H_199_), LBD1- ΔR4 (Q_1_-D_153_). The primers used for constructions were shown in Table 1. PCR conditions were set as follows: 95°C for 5 min, then 30 cycles at 95°C for 45 s, 58°C for 45 s, 72°C for 1 min, and final extension at 72°C for 10 min. PCR products were ligated into pEASY-T1 simple vector (Transgen Biotech, China) and subsequently transformed into *E*. *coli* Trans1-T1 competent cells. Positive clone verified by double digestion was further sequenced by Sangon Biotech (China) to ensure the construction was correct. Wild type LBD1 was inserted into pCold-SUMO vector (Takara, Japan) and the mutants were inserted into pET-32M∙3C vector, respectively.

**Table 1:** All primers used to construct wild type and truncated mutants of LBD1.

*LBD1* and mutants		Directions	Primer sequences
***LBD1***		Forward	5'-CGCGGATCCCAGTTCGTTGACGAAATGCAA-3'
		Reverse	5'-CGCAAGCTTCTAGTGCCAGTTGGCGCAGAAG-3'
***LBD1-ΔR1***		Forward	5'-CGAGGATCCTGTAACGAGACCCACCAGT-3'
		Reverse	5'-CGACTCGAGTTAGTGCCAGTTGGCGC-3'
***LBD1-ΔR2***	**R1**	Forward	5'-CGCGGATCCCAGTTCGTTGACGAAATGCAAGTCTACG-3'
		Reverse	5'-GCACAGGAAACCTTTGCAGAACTGAGCGTCTGGC-3'
	**R3-R4**	Forward	5'-GCCAGACGCTCAGTTCTGCAAAGGTTTCCTGTGC-3'
		Reverse	5'-CCGCTCGAGTTAGTGCCAGTTGGCGCAGAAGGGT-3'
***LBD1-ΔR3***	**R1-R2**	Forward	5'-CGCGGATCCCAGTTCGTTGACGAAATGCAAGTCTACGAG-3'
		Reverse	5'-CTCCTAGCCACTCCGGGCATTTACCAGTAGCGAC-3'
	**R4**	Forward	5'-GTCGCTACTGGTAAATGCCCGGAGTGGCTAGGAG-3'
		Reverse	5'-CGCAAGCTTCTAGTGCCAGTTGGCGCAGAAG-3'
***LBD1-ΔR4***		Forward	5'-CGAGGATCCCAGTTCGTTGACGAAATGC-3'
		Reverse	5'-CGACTCGAGTTAATCCGATCCGCTTAGCAT-3'

### Expression and purification of LBD1 and its mutants

The recombinant pCold-SUMO-LBD1 plasmid was transformed into TransB (DE3) competent cells (TransGen Biotech, China) to induce the expression of His_6_-SUMO-LBD1 with 0.1 mM isopropyl β-D-1-thiogalactopyranoside (IPTG) for 18 h at 16°C in the presence of 0.5 mM CaCl_2_ in LB medium. His_6_-SUMO-LBD1 was purified using prepacked Ni-NTA affinity column (GE Healthcare, USA). Phenylmethanesulfonyl fluoride (PMSF) (Sigma, USA) was applied during the entire purification process to inhibit the protease activity. Purified His_6_-SUMO-LBD1 was subsequently desalted with HiPrep 26/10 column (GE Healthcare, USA) to remove extra imidazole. His_6_-SUMO tag was separated from LBD1 by Ni-NTA column followed the treatment of SUMO protease (20 U/μL) at 4°C for 16 h. Purified LBD1 was analyzed by 12.5% SDS-PAGE and Superdex 200 (GE Healthcare, USA) to determine its molecular weight and conformation in solution. LBD1 was concentrated *via* ultrafiltration and its final concentration was determined by BCA kit (Beyotime, China). The truncated mutants were expressed and purified as a similar purification procedure of the wild type LBD1 except His_6_-Trx tag of pET-32M∙3C vector was cleaved from LBD1 mutants by PreScission protease (GE Healthcare, USA).

### Pull-down assay

About 0.5 ml Ni-NTA agarose bead was incubated with 2 mg His_6_-SUMO-LBD1 at room temperature for half an hour, and then incubated with 5 mL fresh silkworm larval hemolymph at 4°C for 2 h. Thereafter, the beads were centrifuged and washed five times with binding buffer (20 mM Tris-HCl, pH 8.0, 500 mM NaCl). Finally, the beads were eluted sequentially with binding buffer containing 80, 100, 250 and 500 mM imidazole, respectively. All the eluted fractions were analyzed by 12.5% SDS-PAGE to analyze the protein band binding with LBD1. His_6_-SUMO-LBD1, the hemolymph, SP1 and SP2 were set as the control.

### Mass spectrometry analysis

The appropriate band identified to bind with LBD1 was excised from SDS-PAGE gel, placed into a clean microcentrifuge tube, and then digested with trypsin as described [47]. The digested band was run on a MALDI-TOF/TOF 5800 mass spectrometry (AB SCIEX, USA) to obtain the corresponding peptide mass fingerprinting (PMF) using GPS Explorer 3.5 (Applied Biosystems, USA) and Mascot (Matrix Science, UK). PMF data were further indexed using Andromeda search engine to assign the corresponding peptide sequences in an integrated silkworm proteome database [48, 49].

### Purification of *Bombyx mori* storage proteins

*Bombyx mori* storage proteins were separated and purified as previously described [12, 47]. Fresh silkworm hemolymph was collected from female larvae on day 7 of the fifth instar and then centrifuged at 12,000 g at 4°C for 15 min. The supernatant was collected and placed into a water bath at 76°C for 10 min, and then centrifuged at 12,000 g at 4°C for 20 min. Subsequently, the supernatant was filtered through 0.45 μm membrane, and then precipitated by saturated (NH_4_)_2_SO_4_ with an increasing concentration at 4°C. The precipitate was separated from supernatant by centrifugation at 16,000 g for 20 min. Each precipitate was dissolved in 20 mM Tris-HCl, pH 8.0, 500 mM NaCl and analyzed with the final supernatant by 10% SDS-PAGE. Purified storage protein was desalted to remove extra (NH_4_)_2_SO_4_ and the molecular conformation was further analyzed by Superdex 200. Protein concentration was determined as previously described.

### Western and far western blotting assay

About 5 μg pure SP1 and SP2 were denatured, separated by 10% SDS-PAGE and then electrotransferred to polyvinylidene difluoride (PVDF) membrane (Roche, Swiss) at 25 V for 30 min by Trans-Blot Turbo (Bio-rad, USA). The PVDF membranes were then blocked in 5% (v/v) skim milk for 2 h at room temperature and incubated with a 1:10,000 dilution of rabbit anti-SP1 and anti-SP2 at 4°C overnight, respectively. After three rinses with TBST buffer (20 mM Tris-HCl, pH 8.0, 150 mM NaCl and 0.1% Tween-20), PVDF membranes were incubated with goat anti-rabbit HRP-conjugated IgG at 37°C for 1 h. The blotting signal was finally detected with an enhanced chemiluminescence (ECL) western blotting kit (Thermo Fisher, USA).

Far western blotting was performed as described [50]. About 1 μg pure SP1 and SP2 were separated by 10% SDS-PAGE and then transferred to PVDF membrane. Bovine serum albumin (BSA) was set as a control. Subsequently, denatured BSA, SP1 and SP2 were renatured on the membrane. Then the membrane was blocked and incubated with 30 μg pure LBD1. The standard western blotting was applied in the following procedure to analyze the interactions between LBD1, SP1 and SP2.

### Immunoprecipitation assay

About 5 μg LBD1 antibody was diluted into 500 μL washing buffer (20 mM Tris-HCl, pH 8.0, 150 mM NaCl, 5% glycerol), then added into 20 μL 5% (w/v) BSA-blocked protein A/G PLUS-agarose beads and incubated at 4°C for 30 min. The mixtures of agarose beads-anti-LBD1 were centrifuged, and then washed four times with 1 mL washing buffer. LBD1 was first incubated with SP1 and SP2, respectively, then incubated with anti-LBD1 conjugated agarose beads overnight. The mixtures were centrifuged to separate the precipitate and supernatant. The precipitates were washed five times with washing buffer and then analyzed by a standard western blotting procedure.

### Isothermal titration calorimetry (ITC) assay

The binding of SP1/SP2 with LBD1 and its mutants were performed in 20 mM Tris-HCl, pH 8.0, 150 mM NaCl at 25°C on a MicroCal VP-ITC (GE Healthcare, USA). LBD1, SP1 and SP2 were freshly prepared and exchanged into the buffer prior to ITC titration experiment. LBD1 or its mutants were placed into the stirred cell as receptors, and SP1/SP2 was loaded into the syringe as a ligand. SP1/SP2 concentration was 210 μM, and LBD1 and its mutants concentrations were 16.9~20 μM, respectively. The procedure for ITC experiment was set as: an initial injection of 2 μL ligand, followed by 10 μL injection of ligand at 240 s interval until the complete saturation of all binding sites had been achieved. Raw ITC data were recorded and the dilution and mixing heats of SP1/SP2 were subtracted in each dataset of experiments according to the final baseline of ITC titration curve. Prior to peak integration, the binding reaction heats were normalized as a function of ligand concentration. Each measurement was repeated at least three times. The data were fitted as a single-site binding model by ORIGIN 7.0 (OriginLab Corp., USA) [51].

## Results and Discussion

### Bioinformatical analysis

**Fig 1:**
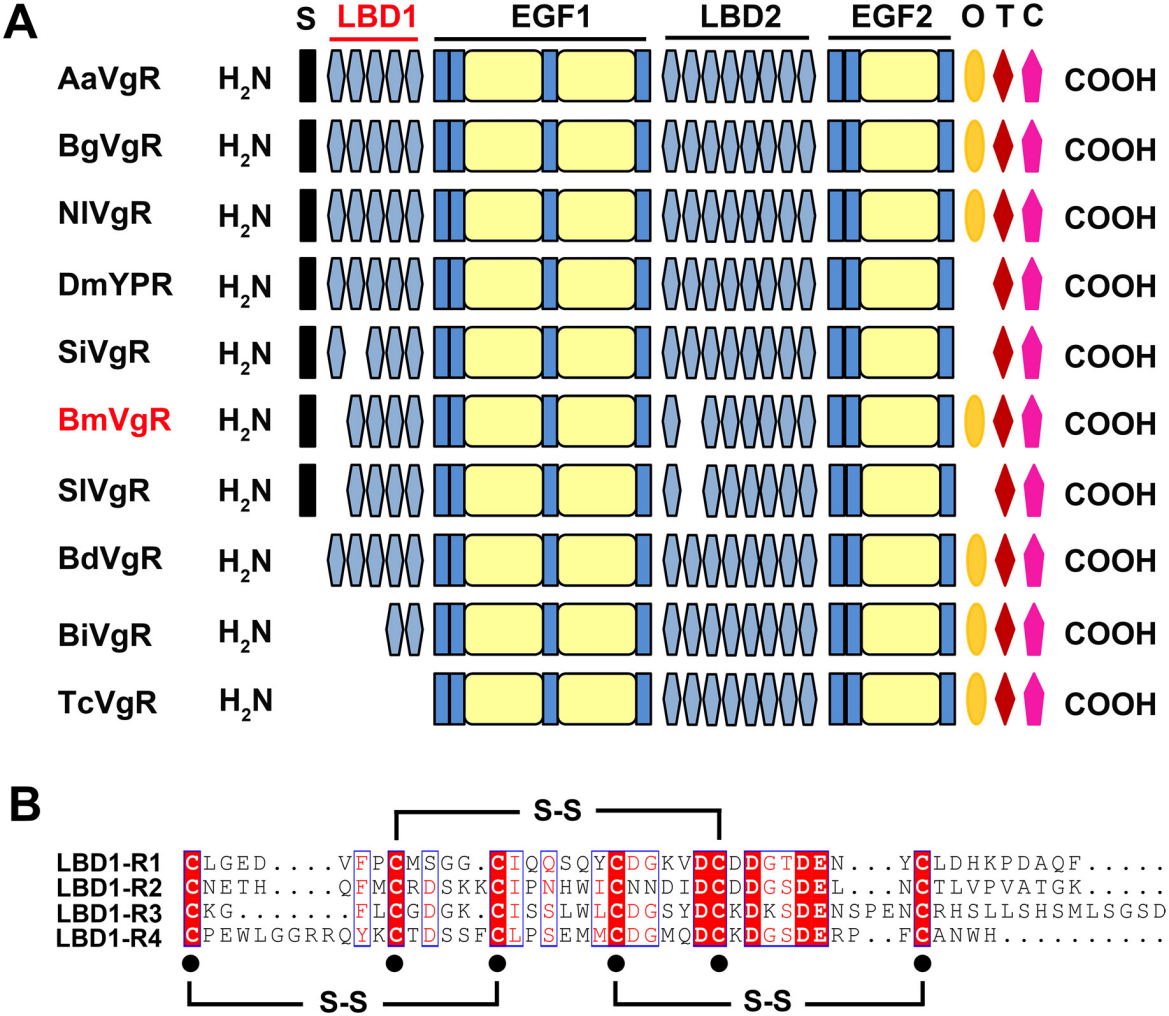
Structural analysis of BmVgR and multiple sequence alignment of LBRs. A. Schematic alignment of modular domains in different insect VgRs. S, signal peptide; LBD, ligand binding domain; class A, Cys-rich repeats; EGF, epidermal growth factor; class B, Cys-rich repeats; O, O-linked sugar domain; T, transmembrane domain; C, cytoplasmic domain. AaVgR (*Aedes aegypti* VgR, AAK15810), BgVgR (*Blattella germanica* VgR, CAJ19121), NlVgR (*Nilaparvata lugens*, ADE34166), DmYPR (*Drosophila melanogaster* YPR, AAB60217), SiVgR (*Solenopsis invicta* VgR, AAP92450), BmVgR (*Bombyx mori* VgR, ADK94452), SlVgR (*Spodoptera litura* VgR, ADK94033), BdVgR (*Bactrocera dorsalis*, ALA27368), BiVgR (*Bombus impatiens* VgR, XP_003489577), TcVgR (*Tribolium castaneum* VgR, XP_968903). B. Multiple sequence alignment of LBRs.

BmVgR has five modular domains, which includes two LBDs, LBD1 and LBD2. LBD1 is composed of four class A repeats, and LBD2 has seven class A repeats. The numbers and the arrangement of repeats in LBD1 and LBD2 of BmVgR were similar to that of SlVgR, but different from that of other insect species (Fig 1A), which may imply the diversity of VgR structure and function in insects. Multiple sequence alignments showed the amino acid sequences were much conserved between LBRs. Each LBR of LBD1 is composed of 6 cysteine residues, which pair in the way of CI-CIII, CII-CV and CIV-CVI to form three disulfide bonds (Fig 1B).

### Expression and purification of LBD1

Expression system and reducing agent such as DTT may affect the proper pairing of multiple cysteine residues. Calcium ion was essential for the proper folding of LBD as it was able to stabilize the conformation of LBD in solution. In order to express LBD1 with proper conformation, we transformed the recombinant plasmid into TransB (DE3) cells, and investigated the effects of various factors on the expression and conformation of LBD1. The results suggested cysteine-rich LBD1 probably formed false disulfide bonds in the absence of DTT, which resulted in the formation of a large amount of aggregated oligomers (S1A Fig). While adding DTT into the buffer, it facilitated the proper pairing of cysteine residues and thus increased the ratio of the properly folded LBD1 (S1B Fig). In the presence of DTT, calcium ion was added into the buffer, which further stabilized the conformation of LBD1 and decreased the ratio of misfolded LBD1 (S1B and S1C Fig). Furthermore, while adding calcium ion into the medium, it better promoted the proper folding of LBD1 and stabilized the conformation of LBD1 (S1D Fig). These results strongly suggested calcium ion and DTT were essential for the proper folding and conformation of LBD1, which was consistent well with previous researches [28, 29].

**Fig 2:**
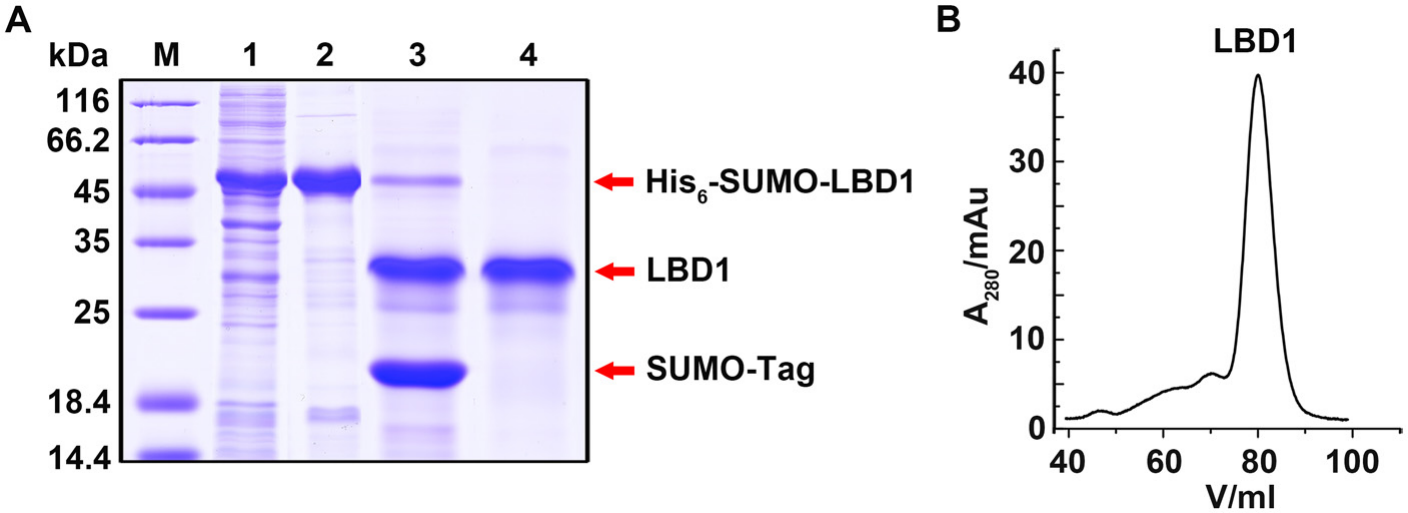
Purification and characterization of LBD1. A. Purification of LBD1. M, protein molecular weight marker; Lane 1, supernatant; Lane 2, His_6_-SUMO-LBD1; Lane 3, His_6_-SUMO-LBD1 cleaved by SUMO protease; Lane 4, purified LBD1. B. Gel filtration analysis of LBD1 conformation.

The recombinant His_6_-SUMO-LBD1 was purified as described. His_6_-SUMO tag was cleaved by SUMO protease and separated from LBD1 by Ni-NTA affinity column (Fig 2A). The conformation of purified LBD1 was analyzed by gel filtration chromatography. The symmetrical and sharp peak of the gel-filtration chromatography indicated that the purified LBD1 had a proper conformation in solution (Fig 2B).

### Identifying the binding of LBD1 and SP1 *via* pull-down assay and mass spectrometry

**Fig 3:**
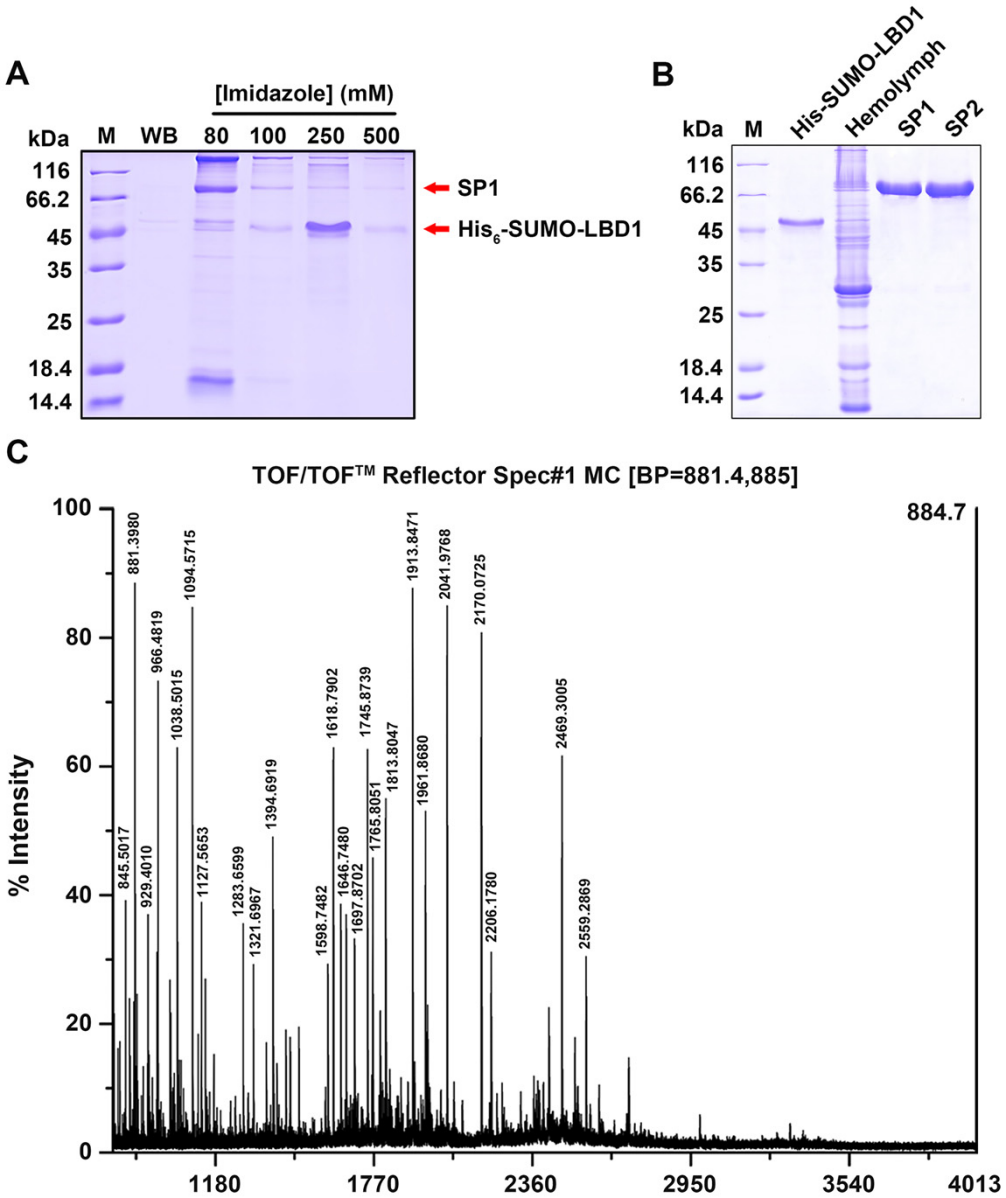
Identifying the binding of LBD1 and SP1. A. Pull-down assay of *Bombyx mori* hemolymph with His_6_-SUMO-LBD1 as a bait. M, protein molecular marker; WB, the elution with washing buffer; 80–250, the elutions with 80–250 mM imidazole. The red arrow above and below indicated the band of SP1 and His_6_-SUMO-LBD1, respectively. B. SDS-PAGE of His_6_-SUMO-LBD1, *Bombyx mori* hemolymph, pure SP1 and SP2. C. PMF of the excised band from SDS-PAGE gel identified by MALDI-TOF/TOF MS.

Purified His_6_-SUMO-LBD1 was used to capture the binding partner from *Bombyx mori* hemolymph *via* pull-down assay. SDS-PAGE gel analysis showed a protein band with a molecular weight between 66.2 kDa and 116 kDa, as indicated by the arrowhead in the elution of 80–500 mM imidazole (Fig 3A). This band was excised from SDS-PAGE gel and further identified by MALDI-TOF/TOF mass spectrometry (MS) (Fig 3C). MS analysis indicated that the PMF of this band could be assigned to the accession ID of BGIBMGA011266 in the silkworm genome database (SilkDB), which represented the female-specific storage protein SP1 of *Bombyx mori* with a molecular mass of about 87 kDa (Table 2).

**Table 2:** Peptide sequences identified by MALDI-TOF/TOF MS.

Name	Accession No. in SilkDB	Coverage(%)	MW/pI	Quality Peptide	Peptide Sequences
**SP1**	BGIBMGA011266	23.56	87255.5/6.78	1037.518	EQFSFPGVK
				1393.724	EQFSFPGVKVEK
				1013.5	FADVMIYR
				1141.595	FADVMIYRK
				2606.327	LNHHPFQVSIDVMSDKTVDAVVR
				1665.823	VLYYAKDFDVFMR
				2041.016	YYGITVTDDNLVVIDWR
				2169.111	YYGITVTDDNLVVIDWRK
				2516.357	LMLPLGTIGGLEMQMYVIVSPVR
				1960.908	INGGMFVYAFTAACFHR
				1792.908	RGEIMMYANQQLLAR
				1960.92	GETFVHTNELQMEEAVK
				1812.835	SSMDMQGFIPEYLSTR
				2468.354	LMLPLGTIGGLEMQMYVIVSPVR
				1693.784	RLDMFELDSFMYK

### Purification and characterization of SP1 and SP2

**Fig 4:**
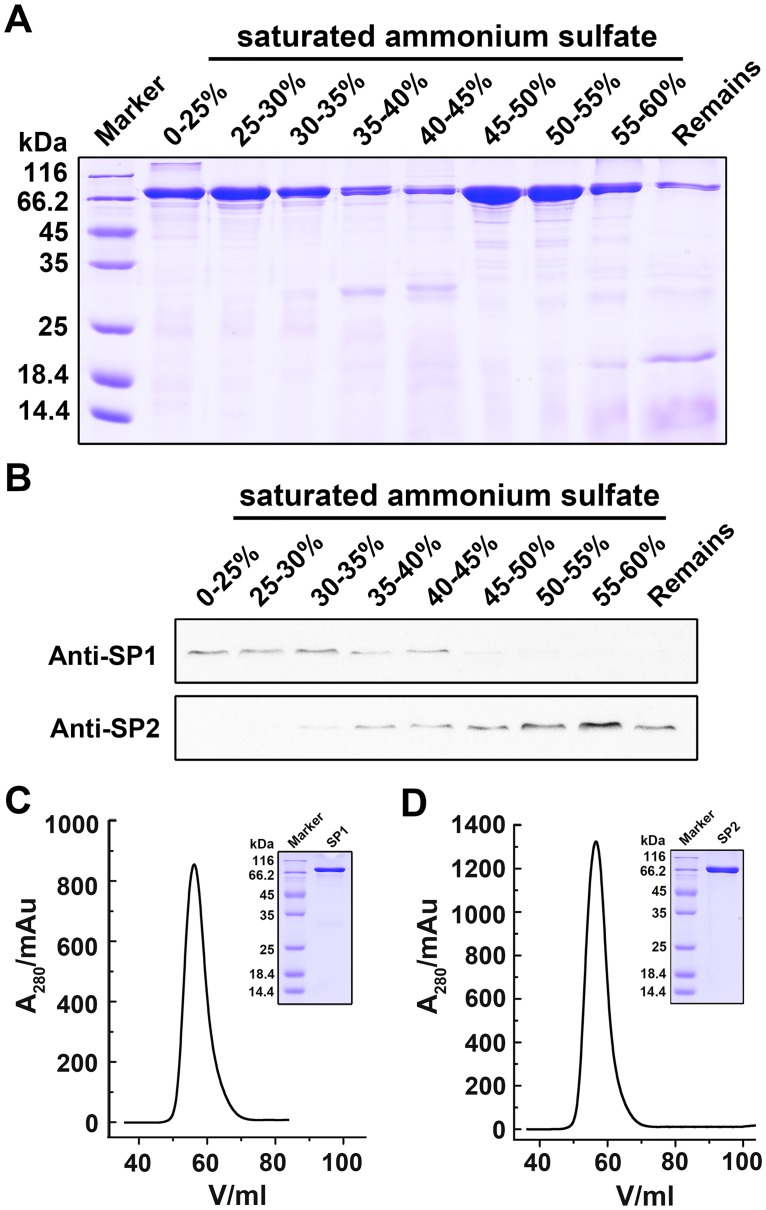
Purification of SP1 and SP2 from silkworm hemolymph. A. SDS-PAGE analysis of the precipitates by different fractions of saturated ammonium sulfate. B. Western blotting analysis of the precipitates with anti-SP1 and anti-SP2. C-D. Gel filtration analysis of SP1 (C) and SP2 (D) conformations in solution.

SP1 and SP2 were purified from silkworm hemolymph followed heat treatment, saturated ammonium sulfate fractional precipitation and gel filtration chromatography as described. SDS-PAGE analysis showed SP1 and SP2 in the hemolymph could be precipitated by various fractions of saturated ammonium sulfate (Fig 4A). Western blotting assay further identified SP1 and SP2 in each precipitate with specific anti-SP1 and anti-SP2 as antibodies. The result showed that most of methionine-rich SP1 could be precipitated by 0–45% saturated ammonium sulfate, however, aromatic-rich SP2 could only be precipitated by 30–60% saturated ammonium sulfate (Fig 4B). In the precipitate of 0–30% saturated ammonium sulfate, SP2 was not detectable on the PVDF membrane. Therefore, this fraction was further used to purify SP1 *via* gel filtration chromatography. Similarly, SP1 was not detectable in the precipitate of 55–60% saturated ammonium sulfate, which thus was used to purify SP2 in the following procedure. Gel filtration analysis showed the purified SP1 and SP2 were in proper conformational states as indicated by the symmetrical and sharp peaks of chromatography (Fig 4C and 4D).

### LBD1 specifically binds with SP1 rather than SP2

**Fig 5:**
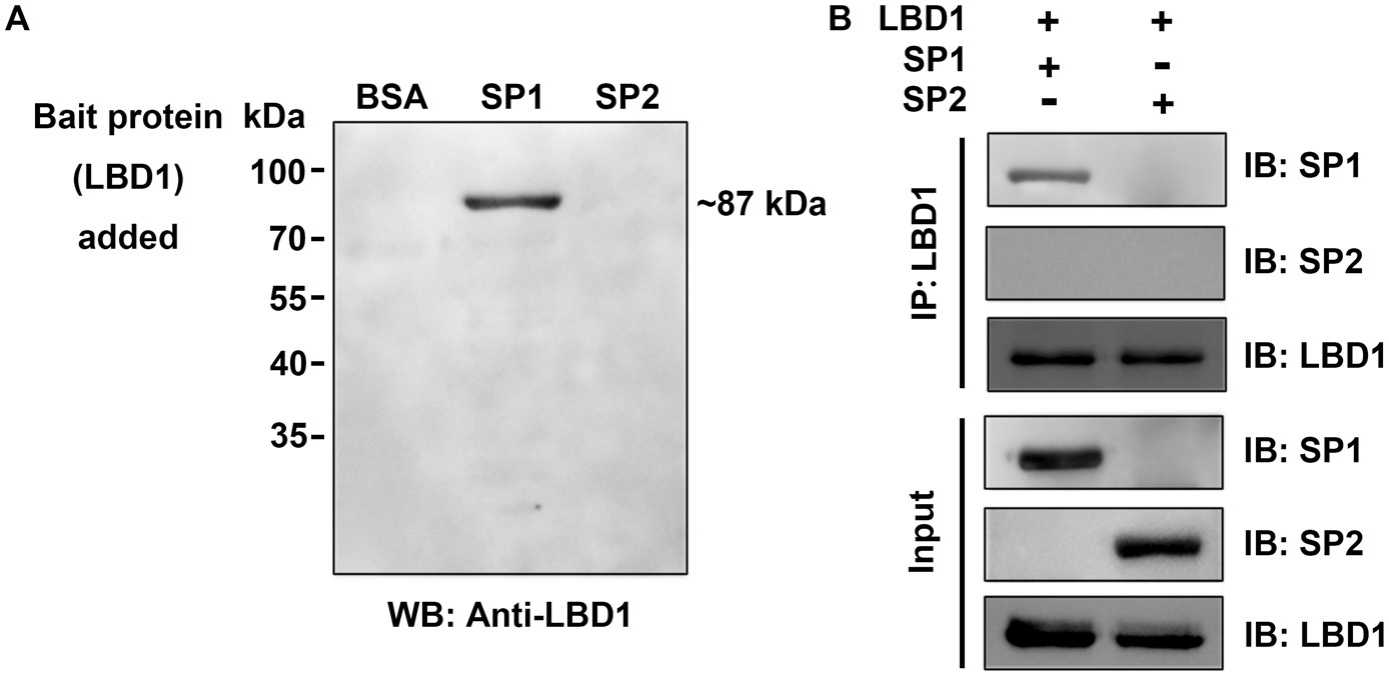
Interaction analysis of LBD1 and SP1/SP2. A. Far western blotting assay. B. Immunoprecipitation assay.

Previous pull-down assay and MS analysis had revealed the interaction of LBD1 and SP1. To further elucidate whether the binding of LBD1 with SP1 was specific, far western blotting assay was carried out to analyze the binding of LBD1 with SP1 and SP2. BSA was used as a control. The results showed that LBD1 could bind with the female-specific SP1, but not BSA and SP2 on the PVDF membrane (Fig 5A). In addition, immunoprecipitation assay was performed to verify the interaction between LBD1 and storage proteins. The results showed that LBD1 specifically bound with SP1 rather than SP2 (Fig 5B), which was consistent well with the previous finding from pull-down assay and MS analysis as well as far western blotting assay.

**Fig 6:**
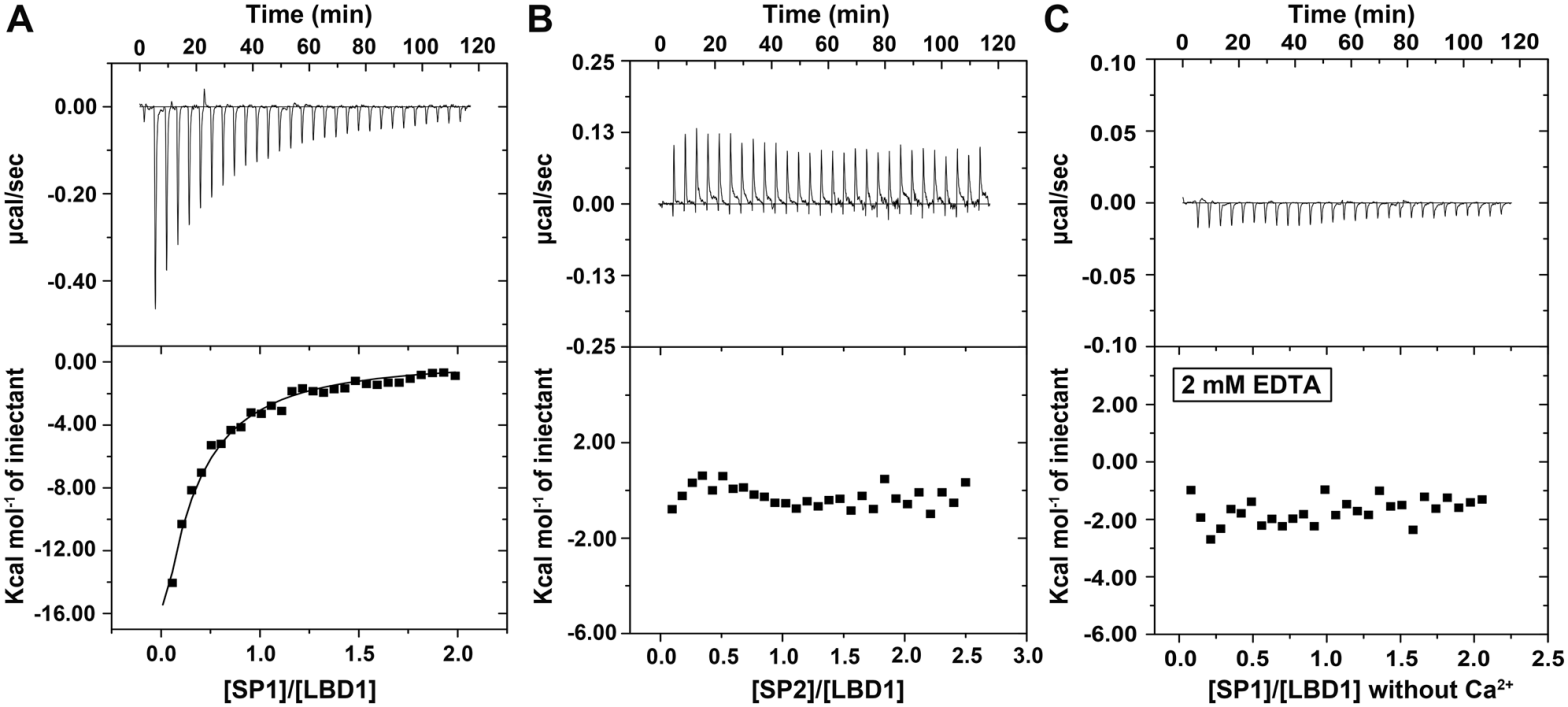
ITC assay of the binding of LBD1 with SP1/SP2. The thermogram and binding isotherm of ITC experiments were shown in the upper and lower panel, respectively. A-B. The titrations of SP1 (A) and SP2 (B) into LBD1. C. The titration of SP1 into LBD1 in the absence of Ca^2+^.

To better characterize the direct interaction of LBD1 with SP1/SP2 in solution, ITC experiment was performed. The exothermic reaction could be easily observed during a successive titration of SP1 into LBD1. As the increasing of the molar ratio of SP1/LBD1, the reaction heat decreased gradually until it reached a saturation (Fig 6A). The result indicated LBD1 could bind with SP1 directly in solution, and most of the binding sites of LBD1 had been saturated by SP1 during the stage of saturated titration. However, no obvious reaction heat could be observed during the titration of SP2 into LBD1 (Fig 6B), suggesting LBD1 did not interact with SP2 in solution. This result further confirmed the conclusion drawn by previous observations that LBD1 could specifically bind with the female-specific SP1 rather than SP2.

SP1 is regarded as a female-specific storage protein as SP1 is mainly detected in the hemolymph of female silkworm larva. Only trace amount of SP1 could be detected in the hemolymph of male silkworm larva [12]. It is well known the endocytosis of the female-specific Vg mediated by VgR is vital for the development of the ovary and oogenesis in insects as Vg is able to provide the necessary amino acid and nutrition resource. A previous study has revealed the amino acid composition of SP1 is similar to that of Vg [38]. SP1 is sequestered from the hemolymph into the fat body and then stored as protein granules for pupal tissue formation during the larval-pupal transformation [12]. Since a large amount of storage proteins are released into hemolymph while storage proteins are synthesized in the fat body during the last larval period, non-selective endocytosis alone would not have the ability to import so many proteins into the fat body in a short period, thus the sequestration of storage proteins must be a selective, receptor-mediated process [15]. However, to date, the potential candidates for the specific storage protein receptor on the membrane of the fat body have not been identified in *Bombyx mori*. Previous researches have demonstrated VgR is specifically positioned on the membrane of the ovary in most insect species, however, recently, a non-ovary VgR has been found to be present in the fat body of the oriental fruit fly, *Bactrocera dorsalis* [52], implying VgR may have other unknown function except mediating the endocytosis of Vg in the ovary during the development of insects. It should be noted that before the endocytosis occurs in the ovary, Vg is released into hemolymph from the fat body where it is synthesized. Thus it is reasonable to conclude BmVgR may be present on the cell membrane of the fat body to mediate the transmembrane transport of Vg from the fat body into hemolymph. Our result confirmed LBD1 of BmVgR could specifically bind to the female-specific SP1 rather than SP2. Taking together the above literatures and our result, the specific binding of LB1 and the female-specific SP1 implies BmVgR may mediate the sequestration of SP1 from hemolymph into the fat body during the metamorphosis of *Bombyx mori*.

### Calcium-dependent ligand binding of LBD1 and SP1

For most members of LDLR family, Ca^2+^ could effectively coordinate the proper formation of disulfide bonds and drive the release of lipoprotein [28, 53]. Our study had shown Ca^2+^ was essential for the proper folding and conformation of LBD1 during the expression and purification of LBD1 (S1 Fig). Whether was Ca^2+^ still required for the binding of LBD1 with SP1 once LBD1 had proper conformation with the aid of Ca^2+^? ITC assay was designed to investigate the effect of Ca^2+^ on the binding of LBD1 with SP1. Ca^2+^ bound with LBD1 was stripped by 2 mM EDTA and subsequently removed *via* gel filtration. ITC analysis showed the reaction heat was hardly detectable during the titration of SP1 into LBD1 in the absence of Ca^2+^ (Fig 6C), indicating that Ca^2+^ was essential to maintain and stabilize the proper conformation of LBD1 which was required for the binding of LBD1 with SP1 in solution.

### Roles of LBRs on the binding of LBD1 with SP1

**Fig 7:**
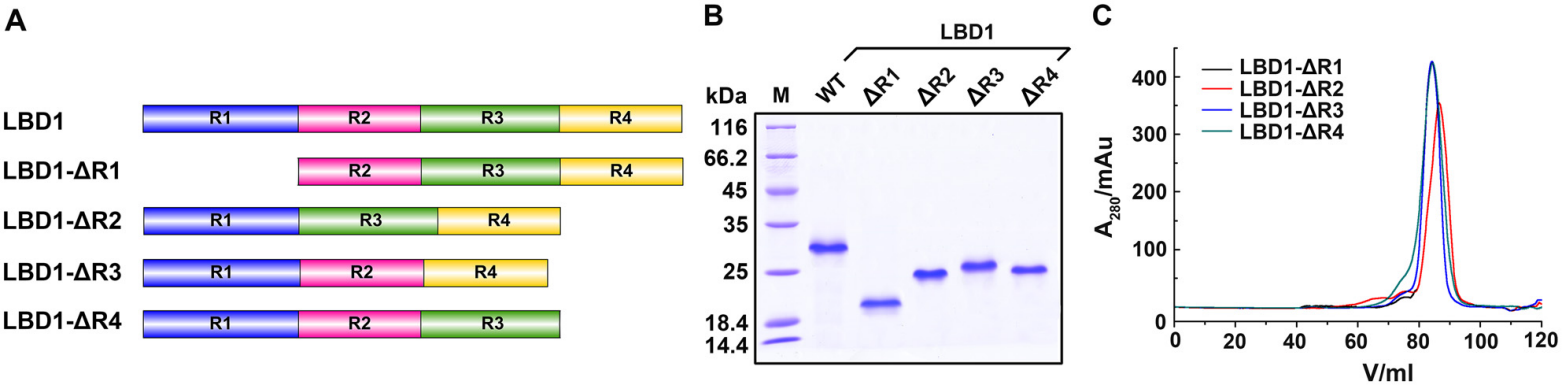
Illustration, purification and characterization of LBD1 mutants with LBR1-4 truncation. A. Schematic illustration of LBD1 and its truncated mutants. B. SDS-PAGE analysis of the purified LBD1 and its mutants. M, protein molecular weight marker; WT, wild type. C. Gel filtration analysis of the conformation of the purified LBD1 mutants.

Compared with vertebrate LDLRs, insect VgRs have an extra LBD with four or five repeats [54, 55]. The differences in the numbers and arrangement of LBRs are responsible for the recognition of diverse ligands [56]. To reveal the roles of LBRs on the binding of LBD1 with SP1, we constructed four LBD1 mutants with LBR1-4 truncation as described, expressed and purified these mutants (Fig 7A and 7B), respectively. Gel filtration analysis showed all LBD1 mutants had proper conformations in solution (Fig 7C), which were good enough for investigating the role of each LBR on the binding of LBD1 with SP1.

**Fig 8:**
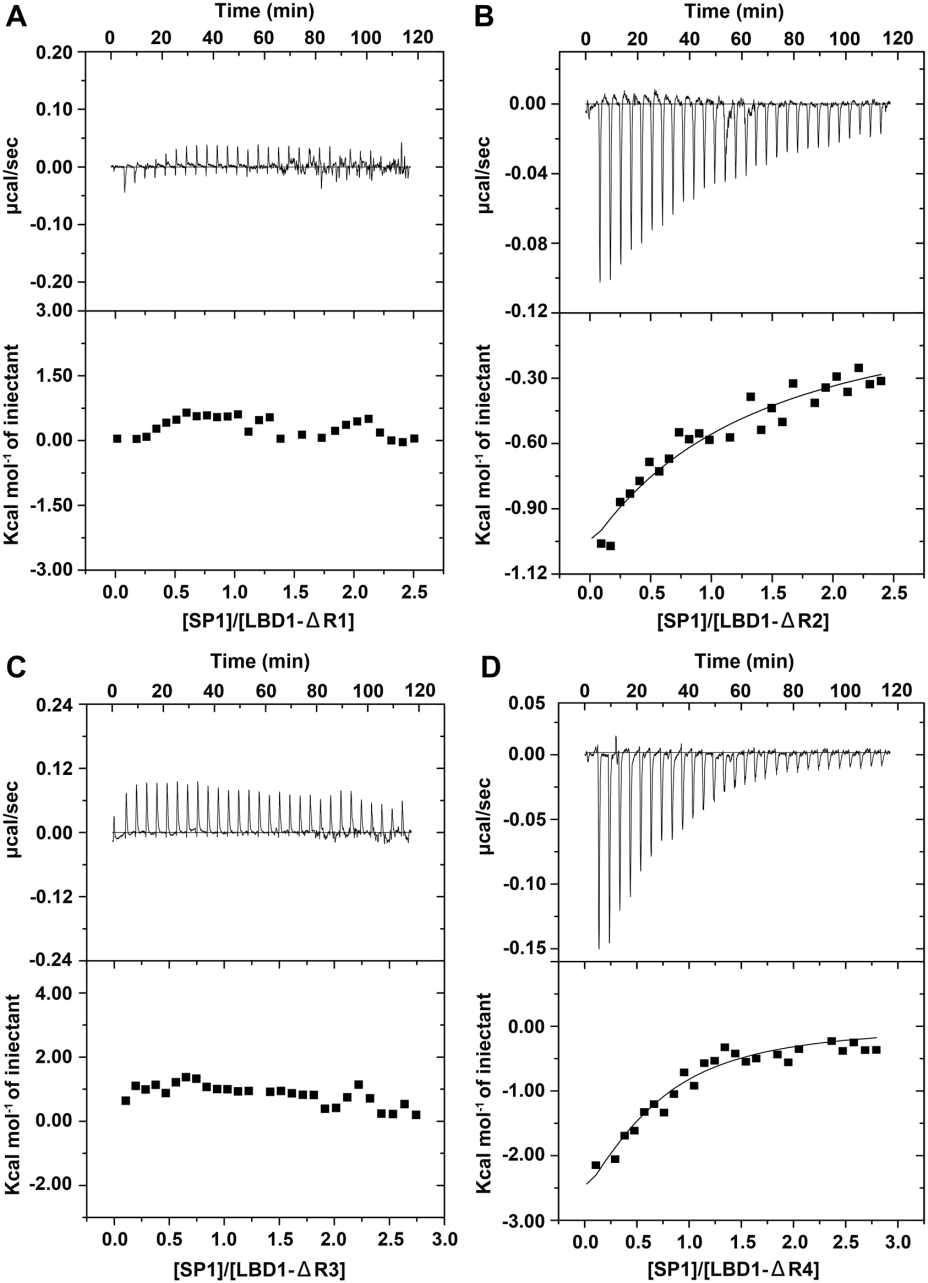
ITC assay of the binding of LBD1 mutants with SP1. A-D. The thermogram and binding isotherm of SP1 titrates into LBD1-ΔR1 (A), LBD1-ΔR2 (B), LBD1-ΔR3 (C) and LBD1-ΔR4 (D).

ITC experiments were performed to analyze the effects of LBRs on the binding of LBD1 with SP1 in solution. The result showed no reaction heat was detected for the titration of SP1 into LBD1-ΔR1 or LBD1-ΔR3 (Fig 8A and 8C), suggesting LBR1 and LBR3 were at least required for the binding of LBD1 with SP1. However, the obvious reaction heat could be observed during the titration of SP1 into LBD1-ΔR2 or LBD1-ΔR4 (Fig 8B and 8D), indicating LBR2 or LBR4 was not necessary for the binding of LBD1 with SP1. While comparing the reaction heat of LBD1-ΔR2 or LBD1-ΔR4 with SP1 to that of LBD1, the result showed that the reaction heat of LBD1-ΔR2 or LBD1-ΔR4 with SP1 was lower than that of LBD1 with SP1 (Figs 6A, 8B and 8D), indicating that the truncation of LBR2 or LBR4 resulted in the affinity decrease of LBD1 with SP1 in solution. Our result suggested although the amino acid sequence has high similarity between LBRs, the role of each LBR was different for the binding of LBD1 with SP1, which was consistent well with previous reports [34, 35].

**Fig 9:**
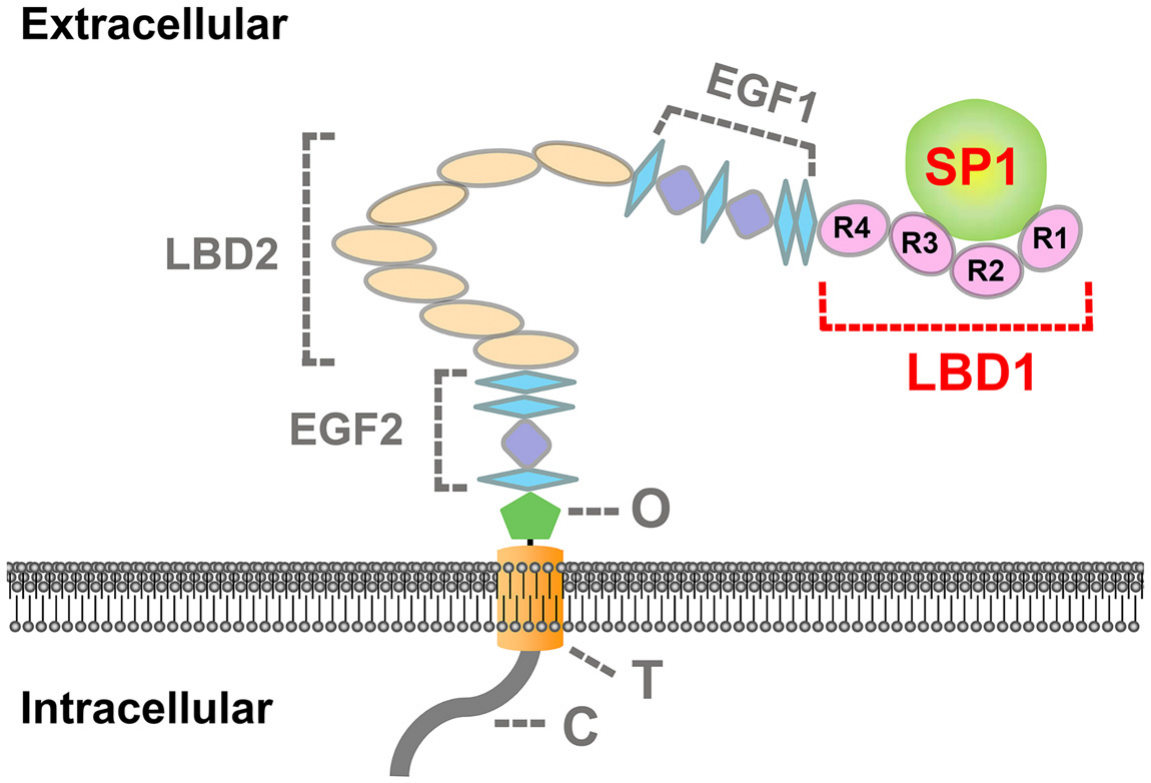
A proposed model of the binding of BmVgR with the female-specific SP1 in *Bombyx mori*. LBD1, the first ligand binding domain; R1-4, ligand binding repeat (LBR)1-4; LBD2, the second ligand binding domain; EGF1, the first epidermal growth factor precursor homology domain; EGF2, the second epidermal growth factor precursor homology domain; O, O-linked sugar domain; T, transmembrane domain; C, cytoplasmic domain.

LDLR family members are able to bind with a large number of ligands. The specificity and multiplicity of LBDs binding with ligands are mainly determined by the numbers and the arrangement of LBRs in LBDs [30–33]. Mutational analyses have shown the binding of apoE with LDLR/vLDLR depends on LBR5, whereas LBRs 2–6 are required for the binding of apoB with LDLR/vLDLR [57]. However, for the binding of RAP with vLDLR, LBRs 1–3 have been demonstrated to be especially important [58, 59]. Also, LBR3 is critical for the binding of RAP with chicken VgR [36]. In our study, ITC analysis suggested LBR1 and LBR3 were essential for the binding of LBD1 and the female-specific SP1, indicating at least two binding sites located in the LBR1 and LBR3 domain were involved in the binding of LBD1 with SP1. Therefore the binding of VgR with female-specific SP1 was likely mediated *via* the first and third repeat LBR1 and LBR3 of LBD1, as illustrated by the proposed model in Fig 9. Although the essential factor and domains have been determined in certain known LDLRs as well as BmVgR for the binding with their ligands, more molecular details such as the key amino acids are still largely unknown. Therefore, expanding our knowledge of the mechanism of LDLRs such as BmVgR interacting with their corresponding ligands will provide signficant contribution towards a better understanding of the relationship between protein structure and function.

As an important amino acid and nutrition resource, it is beyond doubt that the female-specific SP1 plays critical roles for the metamorphosis and oogenesis of insects. Consequently, the sequestration of SP1 from hemolymph into the fat body is a very attractive topic during the development of insects. In this study, we have identified the interaction between the female-specific SP1 and LBD1 of BmVgR and determined the essential factor and domains for the binding of LBD1 and SP1 in detailed biochemical analysis, however, more evidences such as the position and the expression of BmVgR on the fat body before pupation as well as a direct observation of the uptake of SP1 mediated by BmVgR are still required in the future to better reveal the physiological significance behind the specific binding of LBD1 and SP1.

## Conclusion

In this study, we have demonstrated the first ligand binding domain of BmVgR, LBD1, specifically binds to the female-specific storage protein SP1 of *Bombyx mori*, rather than SP2, and Ca^2+^ is essential for the proper conformation of LBD1 as well as the binding of LBD1 with SP1 in detailed biochemical analysis. Our results suggest the first and third ligand binding repeats LBR1 and LBR3 are at least required for the binding of LBD1 with SP1, and LBR2 and LBR4 also make a certain contribution to this specific binding. Our studies imply BmVgR may be involved in the transmembrane transport of the female-specific SP1 from hemolymph into the fat body during the larval-pupal transformation of *Bombyx mori*.

## Supporting Information

S1 Fig Effects of Ca^2+^ and DTT on the conformation of His_6_-SUMO-LBD1. Gel filtration analysis of the purified His_6_-SUMO-LBD1 in the absence of Ca^2+^ and DTT (A), in the presence of 1 mM DTT in the buffer (B), in the presence of 1 mM DTT and 1 mM Ca^2+^ in the buffer (C) and in the presence of 1 mM DTT and 1 mM Ca^2+^ in the buffer and 0.5 mM Ca^2+^ in the medium (D).
(TIF)
