# Supplementary figures and images for: LBD1 of Vitellogenin Receptor Specifically Binds to the Female-Specific Storage Protein SP1 via LBR1 and LBR3
Source: PLoS One. 2016 Sep 16;11(9):e0162317. doi: 10.1371/journal.pone.0162317 (PMC5026343; doi:10.1371/journal.pone.0162317)

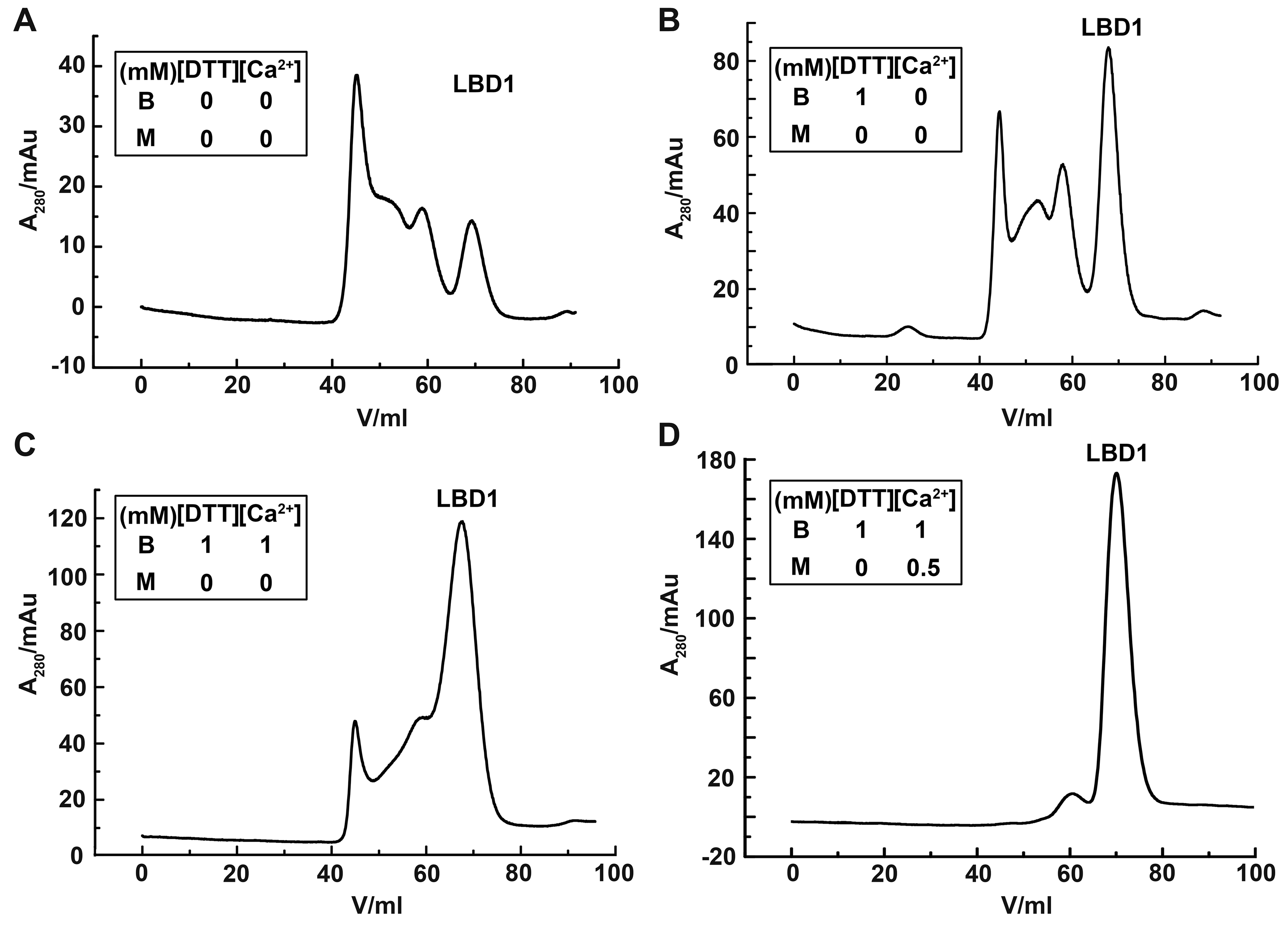

Supplement: S1 Fig — Gel filtration analysis of the purified His6-SUMO-LBD1 in the absence of Ca2+ and DTT (A), in the presence of 1 mM DTT in the buffer (B), in the presence of 1 mM DTT and 1 mM Ca2+ in the buffer (C) and in the presence of 1 mM DTT and 1 mM Ca2+ in the buffer and 0.5 mM Ca2+ in the medium (D). (TIF) [file pone.0162317.s001.tif]
